# Estimating the local spatio‐temporal distribution of malaria from routine health information systems in areas of low health care access and reporting

**DOI:** 10.1186/s12942-021-00262-4

**Published:** 2021-02-12

**Authors:** Elizabeth Hyde, Matthew H. Bonds, Felana A. Ihantamalala, Ann C. Miller, Laura F. Cordier, Benedicte Razafinjato, Herinjaka Andriambolamanana, Marius Randriamanambintsoa, Michele Barry, Jean Claude Andrianirinarison, Mauricette N. Andriamananjara, Andres Garchitorena

**Affiliations:** 1 Stanford University School of Medicine, Stanford, CA USA; 2 Department of Global Health and Social Medicine, Harvard Medical School, Boston, USA; 3 NGO PIVOT, Ranomafana, Madagascar; 4 Direction de La Démographie et des Statistiques Sociales, Institut National de La Statistique, Antananarivo, Madagascar; 5 Center for Innovation in Global Health, Stanford University, Stanford, CA USA; 6 Ministry of Public Health, Antananarivo, Madagascar; 7 National Institute of Public Health, Antananarivo, Madagascar; 8 MIVEGEC, Univ. Montpellier, CNRS, IRD, Montpellier, France

## Abstract

**Background:**

Reliable surveillance systems are essential for identifying disease outbreaks and allocating resources to ensure universal access to diagnostics and treatment for endemic diseases. Yet, most countries with high disease burdens rely entirely on facility-based passive surveillance systems, which miss the vast majority of cases in rural settings with low access to health care. This is especially true for malaria, for which the World Health Organization estimates that routine surveillance detects only 14% of global cases. The goal of this study was to develop a novel method to obtain accurate estimates of disease spatio-temporal incidence at very local scales from routine passive surveillance, less biased by populations' financial and geographic access to care.

**Methods:**

We use a geographically explicit dataset with residences of the 73,022 malaria cases confirmed at health centers in the Ifanadiana District in Madagascar from 2014 to 2017. Malaria incidence was adjusted to account for underreporting due to stock-outs of rapid diagnostic tests and variable access to healthcare. A benchmark multiplier was combined with a health care utilization index obtained from statistical models of non-malaria patients. Variations to the multiplier and several strategies for pooling neighboring communities together were explored to allow for fine-tuning of the final estimates. Separate analyses were carried out for individuals of all ages and for children under five. Cross-validation criteria were developed based on overall incidence, trends in financial and geographical access to health care, and consistency with geographic distribution in a district-representative cohort. The most plausible sets of estimates were then identified based on these criteria.

**Results:**

Passive surveillance was estimated to have missed about 4 in every 5 malaria cases among all individuals and 2 out of every 3 cases among children under five. Adjusted malaria estimates were less biased by differences in populations’ financial and geographic access to care. Average adjusted monthly malaria incidence was nearly four times higher during the high transmission season than during the low transmission season. By gathering patient-level data and removing systematic biases in the dataset, the spatial resolution of passive malaria surveillance was improved over ten-fold. Geographic distribution in the adjusted dataset revealed high transmission clusters in low elevation areas in the northeast and southeast of the district that were stable across seasons and transmission years.

**Conclusions:**

Understanding local disease dynamics from routine passive surveillance data can be a key step towards achieving universal access to diagnostics and treatment. Methods presented here could be scaled-up thanks to the increasing availability of e-health disease surveillance platforms for malaria and other diseases across the developing world.

## Contributions to the literature

- Most countries rely on passive disease surveillance systems, which miss the majority of cases in rural areas of the developing world due to low access to care.
- Precision health mapping has contributed to characterize national and regional heterogeneity in disease burdens, but cannot effectively inform local implementation of disease control activities.
- We present an easily scalable method to obtain accurate estimates of disease spatio-temporal incidence at local scales from passive surveillance, less biased by populations' financial and geographic access to care.
- Our study highlights how digital public health can provide new tools to strengthen local implementation of disease control programs.

## Background

The lack of big data analytics in global health care delivery represents an enormous gap preventing progress toward universal health coverage [1]. The realm of infectious diseases is a prime target for the application of these methods, as increasingly available spatial and temporal information can be harnessed in combination with epidemiological models to produce precise estimates of disease burdens [2, 3]. The most common data sources used to understand burdens of endemic diseases are routine facility-based health management information systems (HMIS) and household surveys. HMIS data have some degree of clinical and temporal granularity and are useful for health planning, but do not provide accurate information on disease burdens because they are only representative of those who access health care. In comparison, nationally representative household surveys (e.g. Demographic and Health Surveys) are heavily relied on for tracking development targets and establishing control priorities, but their data are clinically and spatio-temporally coarse (they are collected every 5 years, in samples that are representative of large regions), and involve limited diagnostic tests. Designated surveillance sites can add high quality data in particular locations, but are expensive and not scalable for localized planning. The prevailing approach for bridging this space is in the form of precision health mapping, where health outputs from coarse epidemiological data are fit from much more granular geospatial environmental data [4–6]. Though this approach produces projections at fine spatio-temporal scales over large geographic areas, these cannot be used by district managers for local planning due to limited accuracy. This represents a significant missed opportunity, because health systems are sitting on enormous quantities of granular data that could be used for local disease control if systematic biases in these data could be addressed.

Malaria is a good example of the challenges and opportunities in the use of health system data for disease control. Despite being preventable and treatable, malaria continues to cause an estimated 228 million infections and 405,000 deaths worldwide each year [7]. Widespread implementation of malaria control measures such as insecticide-treated bed net distribution and indoor residual spraying has resulted in a steady decrease of global incidence, but this trend has recently slowed and even reversed in some areas [8, 9]. Universal access to rapid diagnosis and treatment is a key strategy to reduce the burden of malaria, but access to health care remains stubbornly low in rural areas of Sub-Saharan Africa (SSA) where most of the burden accumulates [9]. In 2017, only one third of African children with fever were brought to a medical provider. Thus, a substantial number of malaria cases were not diagnosed, treated, or included in surveillance statistics [9]. This could be worsened under the current COVID-19 pandemic, which is disrupting supply chains, community health and outreach activities, and could further undermine access to health facilities due to the stigma associated with COVID-19 [10, 11].

Surveillance is critical for both disease control and elimination, and has become one of the three pillars of the Global Technical Strategy for Malaria 2016–2030 [12]. Most malaria control programs rely on passive surveillance systems via case detection at health facilities. Yet, passive surveillance is known to grossly underestimate the incidence of malaria [13–16] because only symptomatic patients who seek care at health facilities are recorded. In 2012, the World Health Organization estimated that only 14% of malaria cases worldwide were detected with routine surveillance [17]. Even in countries committed to malaria elimination, nearly two thirds of cases are missed by national surveillance systems [18]. Passive surveillance is especially unsuited to estimate local malaria burdens for remote populations in rural areas, as health centers are sparsely distributed and health care utilization tends to decrease exponentially as distance to a health facility increases [19–22]. Active surveillance can enhance case detection, but its application remains limited to near-elimination areas due to resource constraints [23]. Thus, innovations are needed to improve the use of passive surveillance data in high transmission areas in order to increase the ability of local control programs to track disease dynamics within a health district, efficiently deploy resources, and target interventions to high-risk populations.

The situation of Madagascar is illustrative of many countries with high burdens of malaria and low rates of diagnosis that could benefit from innovations in passive malaria surveillance. Malaria remains one of the leading causes of mortality in the island [24], with 22.4 of its 25.6 million people living in areas with high transmission [25]. Between 2016 and 2017, the country saw an increase of more than half a million cases [8]. Yet, during that time, only 15.5% of children with reported fever had an RDT done and only 10.1% were treated with an antimalarial [26]. Access to healthcare is particularly low in rural areas of the country, where over three quarters of the population live [27]. In 2014, the Ministry of Health (MoH) partnered with the healthcare NGO PIVOT to strengthen the rural health district of Ifanadiana, located in southeastern Madagascar where malaria transmission is highest [28]. Like most health districts in SSA, data for malaria surveillance in Ifanadiana is aggregated periodically by health centers, so that each data point is representative of a catchment area of about 200 km^2^ and covers a population of approximately 10,000 people. Yet, health center registries systematically record patient geographic information at a much finer resolution, which could be used to greatly improve the capacity of local health systems to target malaria interventions. In support of local malaria control efforts, the goal of this study was to develop a novel method to obtain accurate estimates of disease spatio-temporal incidence at very local scales from routine passive surveillance, less biased by populations' financial and geographic access to care. For this, we used a geographically-explicit patient dataset from the registries of the district’s health centers and we adjusted malaria estimates following a detailed characterization of health care utilization drivers in non-malaria patients.

## Methods

### Study site

**Fig. 1:**
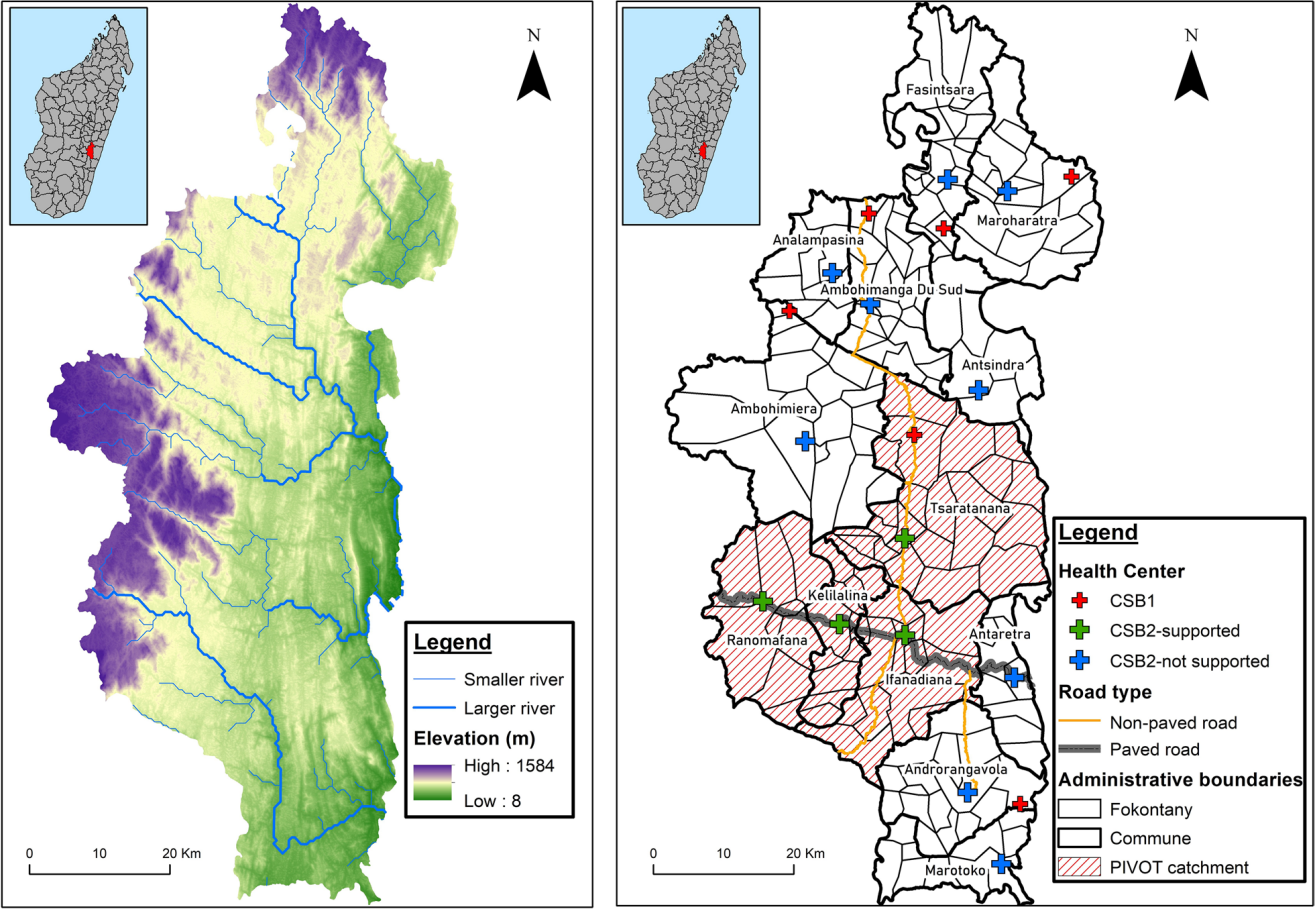
Map of the Ifanadiana district in Madagascar. The left panel shows elevation and waterways. The right panel shows administrative boundaries, roads, health centers (CSBs), and the PIVOT initial catchment area

Ifanadiana is a rural district located in the Vatovavy-Fitovinany region in Madagascar. According to the MoH, Ifanadiana contained approximately 195,000 people in 2015, the vast majority of whom subsist on agriculture (84.8%) [27, 29, 30]. The district is divided into 13 communes (subdivisions with approximately 15,000 people each), which are further divided into 195 Fokontany (the smallest administrative unit, containing one or several villages). Ifanadiana has one reference hospital, one major public health center (CSB2) in each of its 13 communes, and six additional basic health centers (CSB1) in the larger communes (Fig. 1). Passive malaria surveillance is continuously conducted at all of the 19 public health centers throughout the Ifanadiana District, aggregated from routine health registries of clinical patients.

In 2014, a baseline study indicated that Ifanadiana had some of the highest poverty rates and worst health indicators in Madagascar. Nearly three fourths of the population lived in extreme poverty. The mortality rate for children under five was 145 deaths per 1000 live births, more than double the national estimate of 62 per 1000 [29, 31]. Malaria prevalence in the area where the district is located is the highest in the country, with prevalence ranging from 6 to 18% [28]. While more than a third of children under five in Ifanadiana had reported fever in the previous two weeks, only 42% were taken to a health center [32]. Low access to health care was strongly associated with substantial financial and geographic barriers [33]. For instance, only one fourth of the population lives within an hour's travel of a health center [34, 35].

Since 2014, PIVOT has supported the public health system of Ifanadiana at all levels (hospital, health centers and community health workers) guided by the WHO framework for health system strengthening [36]. The intervention initially covered approximately one third of the district’s population. In these areas, PIVOT has helped remove financial barriers to care; improved readiness at health facilities, which includes personnel (quantity of staff and training), supply chain (equipment and consumable), infrastructure, and health management information systems; created an ambulance network; and implemented clinical programs that target tuberculosis, malnutrition and childhood illness through strengthened programs at all levels of care. Following PIVOT's support, the number of cases of malaria diagnosed at health centers in these areas experienced a sudden increase due to rapid improvements in overall health care utilization [33, 37]. To further support local malaria control programs, PIVOT aims to support the MoH to optimize interventions geographically in a context of heterogeneous disease burdens.

### Data collection

Data was obtained from health center registers on all individuals who visited a public health center for an outpatient consultation in Ifanadiana district between January 2014 and December 2017. The data were collected via regular visits to each PHC in the district by PIVOT staff every 3–4 months, in agreement with the head of each PHC and the district medical inspector. This allowed for the creation of a patient-level, de-identified digital database. Information including age, Fokontany of residence, and malaria status of each new patient was entered into an electronic database (follow-up visits were excluded). Health center staff made malaria diagnoses with rapid diagnostic tests (RDTs) for patients presenting with fever, following national guidelines. RDTs used in Madagascar during this period were based on a combination of *Plasmodium falciparum* histidine-rich protein 2 (PfHRP2) and *Plasmodium* lactate dehydrogenase (pan-pLDH) bands.

In addition to health system information, survey data from the IHOPE cohort was used to estimate the geographic distribution of fever prevalence by age group in Ifanadiana [32]. The IHOPE longitudinal cohort study, representative of the population in Ifanadiana district, was initiated in 2014 to understand the evolution of health and socio-economic characteristics as one of the information pillars to create a model health district. It consists of a series of biannual surveys conducted by INSTAT on the same households over time, with questionnaires and methods adapted from the Demographic and Health Surveys and other international surveys. The survey has a two-stage stratified sampling design covering 1,600 households (~ 8000 people) in 80 geographic clusters across the district. Information from the cohort, which was available for 2014 (April–May), 2016 (August–September) and 2018 (April–May), included questions to assess reported fever among children under five years (previous two weeks) and among all household members (previous four weeks).

To obtain per capita estimates, population data for each Fokontany were obtained from the MoH [38]. The population of children under five years old was estimated at 18% of the total population, per the MoH. Data on monthly stocks of RDTs at the end of each month and number of days with RDT stock-outs were obtained from each health center’s monthly report to the district. Use of MoH data for this study was authorized by the Secretary General of the MoH, by the Medical Inspector of Ifanadiana district, and by Harvard’s Institutional Review board (IRB). The IHOPE cohort study was approved by the Madagascar National Ethics Committee and Harvard Medical School IRB.

Finally, we used a geographic information system containing data on locations of all health centers, more than 20,000 km of footpaths, over 100,000 buildings, and nearly 5,000 residential areas in the district. This was obtained following a participatory complete mapping of Ifanadiana in 2018–2019, from very high resolution satellite images available through OpenStreetMap [35]. This data was queried on QGIS via the QuickOSM plugin and was used to estimate shortest path distances between health centers and each Fokontany.

### Data analysis

Patient-level information from each health center was aggregated to estimate per capita utilization rates and malaria incidence per month for each Fokontany in Ifanadiana district. Each Fokontany was then matched with its nearest health center. For this, the shortest path distance between all health centers and Fokontany (average distance among all households in the Fokontany) was estimated via the Open Source Routing Machine (OSRM) engine. In order to obtain more realistic estimates of malaria incidence per Fokontany-month, malaria incidence was adjusted to account for underreporting due to stock-outs of RDTs and variable access to healthcare due to geographic and financial barriers, using methods detailed below.

**Fig. 2:**
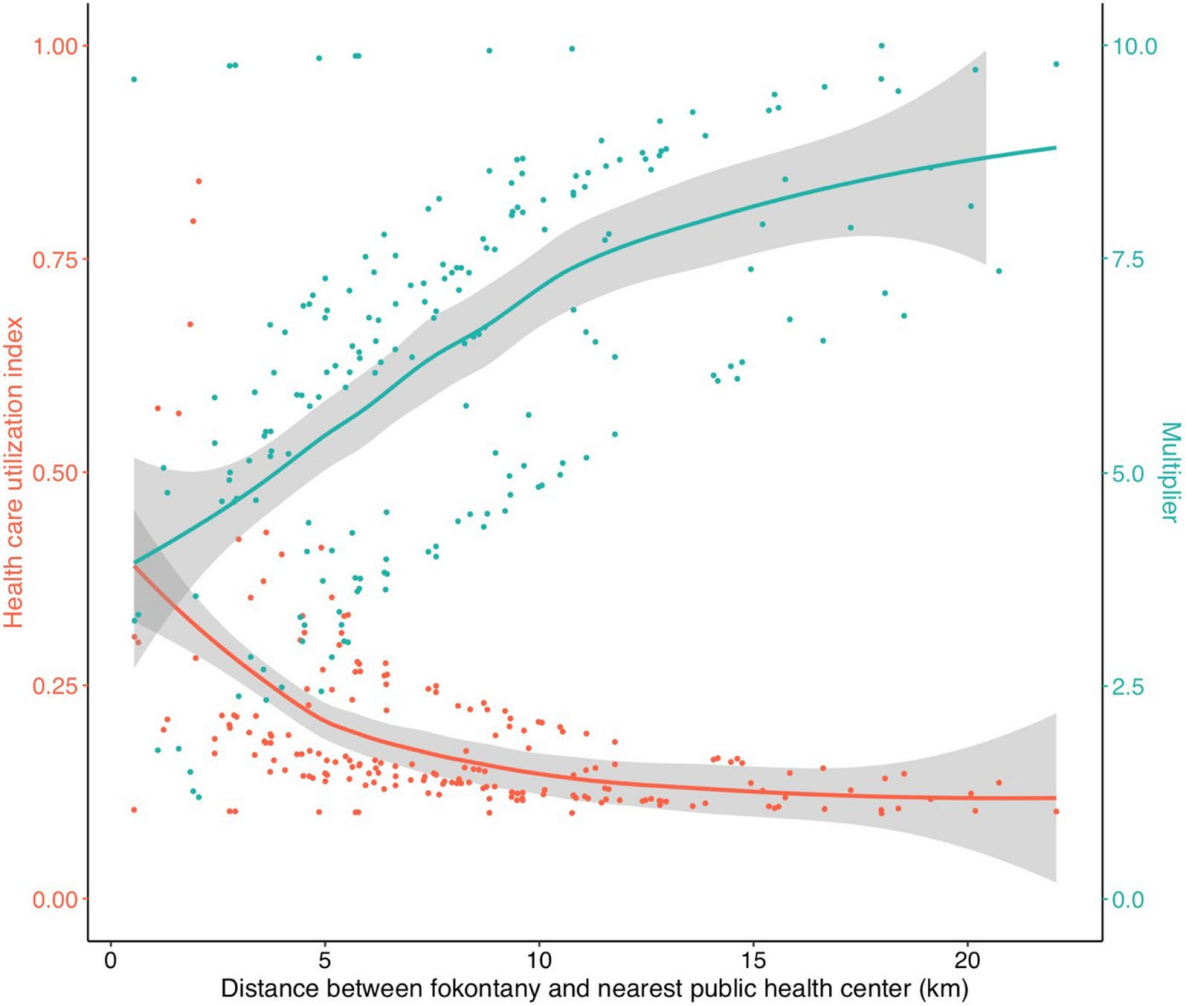
Illustration of benchmark multiplier adjustments to passive malaria surveillance data using a health care utilization index. Each dot represents the average health care utilization index (orange) or resulting multiplier (teal) for one of the 195 Fokontany in Ifanadiana over the study period. In this example, average per capita health care utilization index is normalized from 0.1 to 1, where the maximum is equivalent to 2 visits per year (excluding malaria). Both variables are plotted as a function of distance between each Fokontany and its nearest health center. The solid lines are smoothed conditional means (LOESS method) and the grey areas are the 95% confidence intervals. Fokontany with smaller health care utilization indices have larger multipliers, resulting in greater adjustments after the benchmark multiplier method was applied

A simplified benchmark multiplier method was used to adjust malaria incidence with a health care utilization index produced from non-malaria patients. This method combines information about the known members of a target population (the benchmark; for example, the number of people with malaria who are diagnosed at a health center) with the proportion of the target population that appears in the benchmark (for example, the proportion of people with malaria who go to a health center) [39]. The reciprocal of the proportion is called the multiplier. The true size of the target population (in this case, the true number of people with malaria in Ifanadiana) is estimated as the product of the benchmark and the multiplier. Populations with the best health care access (i.e. located very close to a health center with fee-exemptions in place) are not adjusted, while populations with the worst access are adjusted using the largest multiplier (Fig. 2). We added to this multiplier an index of stock availability to account for patients who would seek care at a health cater but would not get diagnosed due to stock-outs. The simplified benchmark multiplier formula based on these two indices is defined as:

1 $${Mad}_{ij}= \frac{{M}_{ij}}{{U}_{ij}*{S}_{ij}}$$

where *M*_*ij*_ represents the unadjusted monthly malaria cases in fokontany *i* and month *j*, *U*_*ij*_ represents a health care utilization index for the fokontany from the model described below, S_ij_ represents the index of stock availability, which reflects the proportion days in month *j* where the health center matched to fokontany *i* had RDTs in stock. *Mad*_*ij*_ are the resulting adjusted monthly malaria cases in the fokontany *i* for the month *j*. In months in which stock-outs persisted for an entire month at a given health center (*S* = 0; 10 months), we assigned missing values for *Mad*_*ij*_ to all Fokontany served by that health center.

To create *U*_*ij*_ and account for the effect of low health care access on malaria incidence, we used results from a spatio-temporal model of health care utilization in Ifanadiana during the same study period. Details on this model are published elsewhere [34], and coefficients for each variable included in the final model (adjusted odds ratios) are available in Additional file 1: Table S1. Briefly, per capita health center utilization rates for each Fokontany were modeled using Binomial regressions in generalized linear mixed models, with a random intercept introduced for the closest health center. The model accounts for the exponential decrease in utilization as a function of travel distance from each Fokontany to the nearest health center (Additional file 1: Figure S1); the positive impact on consultations of programs implemented to reduce financial and geographic barriers (e.g. user fee exemptions, community health strengthening); the positive linear and seasonal trends in utilization rates in the absence of those programs; baseline differences in the type of health center (lower utilization for CSB1 than CSB2); and the positive association with the number of health staff over time in the closest health center [34]. Based on model predictions of per capita utilization for non-malaria patients, a health center utilization index was produced for each Fokontany-month in Ifanadiana, scaled between zero (no access; set at zero consultations per person-month) and one (full access; set at 0.166 consultations per person-month, equivalent to 2 consultations per person-year, excluding malaria).

Given that the lower limit of the health care utilization index, *U*, can result in drastic changes in magnitude of the resulting adjusted estimates, this was varied from 0.01 to 0.2 in steps of 0.01, with the upper limit remaining one. This range, which limits the multiplier due to the lowest utilization (*1/U*) between 5 and 100, was determined based on exploratory analyses of the effect of the multiplier on malaria incidence, whereby a lower limit above 0.2 had very little effect at adjusting observed biases and a lower limit below 0.01 resulted in an unrealistic overestimation of incidence (e.g. above population size). This allowed for fine-tuning of the adjusted monthly malaria incidence estimates.

Finally, due to extremely low access to care, several Fokontany had no reported malaria cases for several months even during the high transmission season, particularly those located at farther distances (e.g. 10–20 km) from health centers. For instance, 37 of the 195 Fokontany did not have any reported malaria cases during more than half of the high season months (December to May) in the four years of the study, 86% of which were further than 5 km from a health center. Because Fokontany that have a malaria incidence of zero during a given month cannot be adjusted using a multiplier, we explored several strategies to pool the number of malaria cases in these Fokontany with the cases in neighboring Fokontany and estimate a pooled incidence that could then be adjusted for low health care access. We explored pooling with the *k*-nearest neighbors (2, 3, 4 and 5) and with neighbors within a certain distance (3, 4, and 5 km).

The combination of 8 different pooling strategies and 21 different lower limits set for the health utilization index resulted in 168 alternative sets of adjusted malaria incidence estimates, both for individuals of all ages and for children under five.

### Evaluation of model estimates

The lack of a district-representative active surveillance survey during the study period meant that alternative sets of adjusted estimates of malaria incidence from passive surveillance could not be robustly compared to an unbiased training dataset for validation. We established four evaluation criteria to choose the most plausible set of incidence estimates in Ifanadiana based on the available data (Table 1). This was done both for individuals of all ages and for children under 5.

**Table 1:** Evaluation criteria for alternative sets of adjusted malaria incidence estimates

Criteria	Description	Evaluation method
(a) Overall malaria incidence	Overall adjusted malaria incidence for Ifanadiana should be similar to overall malaria incidence in populations with optimal health care access in the district, to avoid under- or overestimation	Ratio of adjusted malaria estimates to malaria in optimal access areas between 0.7 and 1.3
(b) Distance decay	Adjusted malaria incidence estimates should remove the distance decay observed in unadjusted malaria incidence, to limit bias due to geographic access to health care	Ratio of incidence less than 5 km from a health center to incidence greater than 5 km away 0.7–1.3
(c) Financial access	Differences in adjusted malaria incidence between health centers according to fee-exemption status should be minimal, to limit bias due to financial access to health care	Ratio of fee-exemption to no fee-exemption in adjusted malaria estimates between 0.7 and 1.3
((d) Geographic heterogeneity	The geographic distribution of adjusted malaria incidence should be similar to the geographic distribution of fever reported in the IHOPE cohort study during the high transmission season	Percent of hotspot cluster area overlap between the two datasets during high malaria transmission season (SaTScan)

Evaluation criteria are based on: (a) consistency of *overall malaria incidence* in the district with incidence in areas with optimal access to healthcare; (b) reduction of *distance decay* relationship; (c) reduction of bias due to *financial access* to care; and (d) consistency of *geographic heterogeneities* in the district with patterns observed in the IHOPE cohort study. The first three criteria rely on the assumption that the burden of malaria in populations with good access to health care (e.g. those who live near health centers, or in areas where user fees have been removed) is similar to the burden elsewhere because the per capita distribution of malaria is predominantly driven by ecological and epidemiological factors, and not by health care access [40–43]. Although health centers diagnose and treat malaria patients, the main malaria prevention activities in Madagascar (e.g. bed net distribution, indoor residual spraying) that could affect transmission are delivered through mass-campaigns to all at-risk populations.

#### Overall malaria incidence

To avoid under- or overestimation of overall malaria incidence in the district, we assumed that adjusted estimates should be similar to unadjusted malaria incidence in populations with optimal access to health care. These were defined as populations from Fokontany that are in close proximity (≤ 2.5 km) to a PIVOT-supported health center following initial implementation of health system strengthening interventions. These populations travelled short distances to care and benefited from improved facilities, with greater staffing, and point-of-care fees for most health services removed. They represented a total population of 10,583 individuals of all ages, including 1,905 children distributed across 4 Fokontany in 4 communes, with an average health system utilization index of 0.66 (on a scale from 0 to 1). The 4-year annual malaria incidence average in this population for 2014–2017 was 397 cases per 1000 population among individuals (33 cases per 1000 population per month) and 631 cases per 1000 population among children under five (53 cases per 1000 per month). To assess this criterion, we estimated the ratio of average malaria incidence in each adjusted dataset to average malaria incidence in the optimal access dataset. Adjusted datasets with a ratio within 30% of equality (0.7–1.3) were considered most plausible. This first validation allowed variations in the geographic distribution of malaria but set a reasonable reference point for the district average.

#### Distance decay

To limit bias due to geographic access to health care, we assumed that there should not be an exponential distance decay relationship in adjusted malaria incidence (as it was observed in unadjusted incidence estimates, Additional file 1: Figure S1). To assess this criterion, we calculated the ratio of average incidence in Fokontany located fewer than 5 km from a health center to average incidence in Fokontany more than 5 km away. Adjusted datasets with a ratio near 1 (0.7–1.3) were considered as most plausible. This second validation ensured that the geographic distribution of malaria incidence in the adjusted dataset was not associated with heterogeneities in geographic access to health care.

#### Financial access

To limit bias due to financial access to health care, we assumed that average adjusted incidence in the catchment of health centers that implemented user-fee exemptions should be similar to those for which user fees were not in place. Before adjustment, average monthly incidence of malaria among all individuals and children under five *inside* the PIVOT catchment area after financial barriers to care were removed were 13 and 27 per 1000 population, respectively, while the average monthly incidence among all individuals and children under five living *outside* of this area was significantly lower: 6 and 16, respectively (ratio of 2.1 and 1.7). To assess this criterion, we estimated the ratio of average adjusted malaria incidence in the catchment of health centers with user-fee exemptions to health centers without user-fee exemptions. Adjusted datasets with a ratio within 30% of equality (0.7–1.3) were considered as most plausible. This third validation ensured that the temporal and geographic distribution of malaria incidence in the adjusted dataset were not associated with heterogeneities in financial access to health care.

#### Geographic heterogeneity

**Fig. 3:**
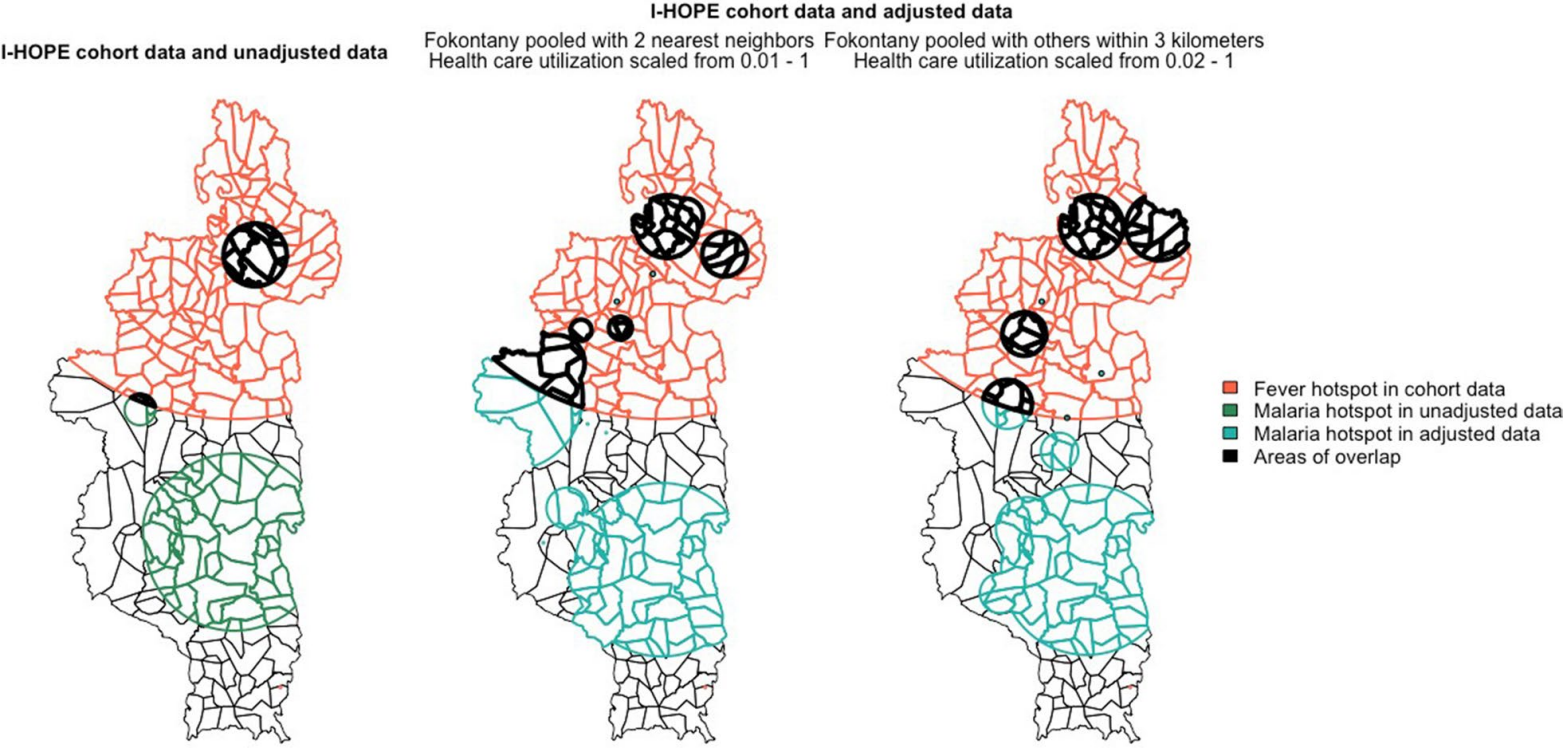
Comparison of geographic hotspots of fever (IHOPE cohort survey) and malaria (health center registers) in Ifanadiana among all individuals during malaria high season, using unadjusted and adjusted estimates. Colored regions represent malaria hotspots in the various data sources and the areas of overlap in bold black. The left panel shows hotspots using unadjusted health center register data, while the center and right panels show examples of hotspots from two of the 168 adjusted datasets. The observed overlap is significantly greater in adjusted datasets, indicating improved consistency between the geographic distribution of fever in the IHOPE cohort study and malaria in health center register data after adjustments

To assess the consistency of heterogeneities in malaria geographic distribution, we assumed that during the high transmission season (December to May) there is a geographic overlap of malaria incidence with the distribution of reported fever in household surveys (April–May). In the high transmission season, 36.6% of individuals of all ages and 41.2% of children under 5 years presenting to health centers had a confirmed malaria diagnosis. Since malaria makes up a high proportion of febrile cases during these periods, we assume that geographic variations in febrile prevalence should be correlated with variations in malaria transmission [44, 45]. To assess this criterion, we estimated average fever prevalence for each of the 80 clusters in the IHOPE cohort as the number of individuals reporting a fever in the previous weeks out of the total number of individuals in the cluster, and we estimated average malaria incidence for each of the 195 Fokontany during the high transmission season. Then, SaTScan software using the Bernoulli spatial model was used to identify geographic clusters of malaria in Ifanadiana district. SaTScan has been used in previous studies to identify spatiotemporal variation of malaria [46] and other illnesses such as diarrheal disease [47], schistosomiasis [48], and colorectal cancer [49]. SaTScan cluster analysis was applied to identify spatial hotspots (i.e. higher than expected by random) among all individuals and among children under five in fever prevalence from survey data, unadjusted malaria incidence from health system data, and each of the adjusted incidence datasets. The area overlapped by geographic hotspots in fever and malaria from these different sources were quantified (Fig. 3).

All analyses were performed with R software, and R packages “lme4,” “gstat,” “rgdal,” “ggplot2,” "rsatscan," "spdep," "sp," "rgeos," "tidyr," and "survey" [50]. Information in this study was reported as per STROBE guidelines (Additional file 2).

## Results

### Malaria incidence in the unadjusted dataset and selection of the most plausible adjustment

Of the 314,443 patients who attended a health center in Ifanadiana district for an outpatient visit between 2014 and 2017, 270,747 patients had a known geographic location and came from within the district. Among these, 73,022 were confirmed malaria cases, 29,124 of which were children under 5 years. Average malaria incidence was 104.6 per 1000 population per year, and varied greatly across seasons. During the high transmission season (December to May), average malaria incidence was 168.0 per 1000 population per month, decreasing during the low transmission season to 41.3 per 1000 per month. There was a clear distance decay in malaria incidence both for individuals of all ages and for children under 5 years (Additional file 1: Figure S1). Table 2 presents summary demographic and geographic characteristics of the patient population and malaria cases that attended one of the 19 health centers.

**Table 2:** Summary statistics of patient population in Ifanadiana health centers, 2014–2017

	Population	Total patients	Total malaria confirmed cases	Malaria incidence per 1000 per year
Total	198,175	270,747	73,022	104.6
Age group				
Under 5 years old	35,672 (0.18)	92,533 (0.34)	29,124 (0.40)	231.8
Over 5 years old	162,504 (0.82)	178,214 (0.66)	43,898 (0.60)	76.7
PIVOT catchment				
Inside	72,152 (0.36)	173,497 (0.64)	42,992 (0.59)	158.0
Outside	126,023 (0.64)	97,250 (0.36)	30,030 (0.41)	70.5
Distance to health center				
0–5 km	63,811 (0.32)	163,656 (0.60)	41,067 (0.56)	186.0
5–10 km	81,787 (0.41)	83,667 (0.31)	25,294 (0.35)	87.9
10–22 km	52,577 (0.27)	23,424 (0.09)	6661 (0.09)	35.2

**Fig. 4:**
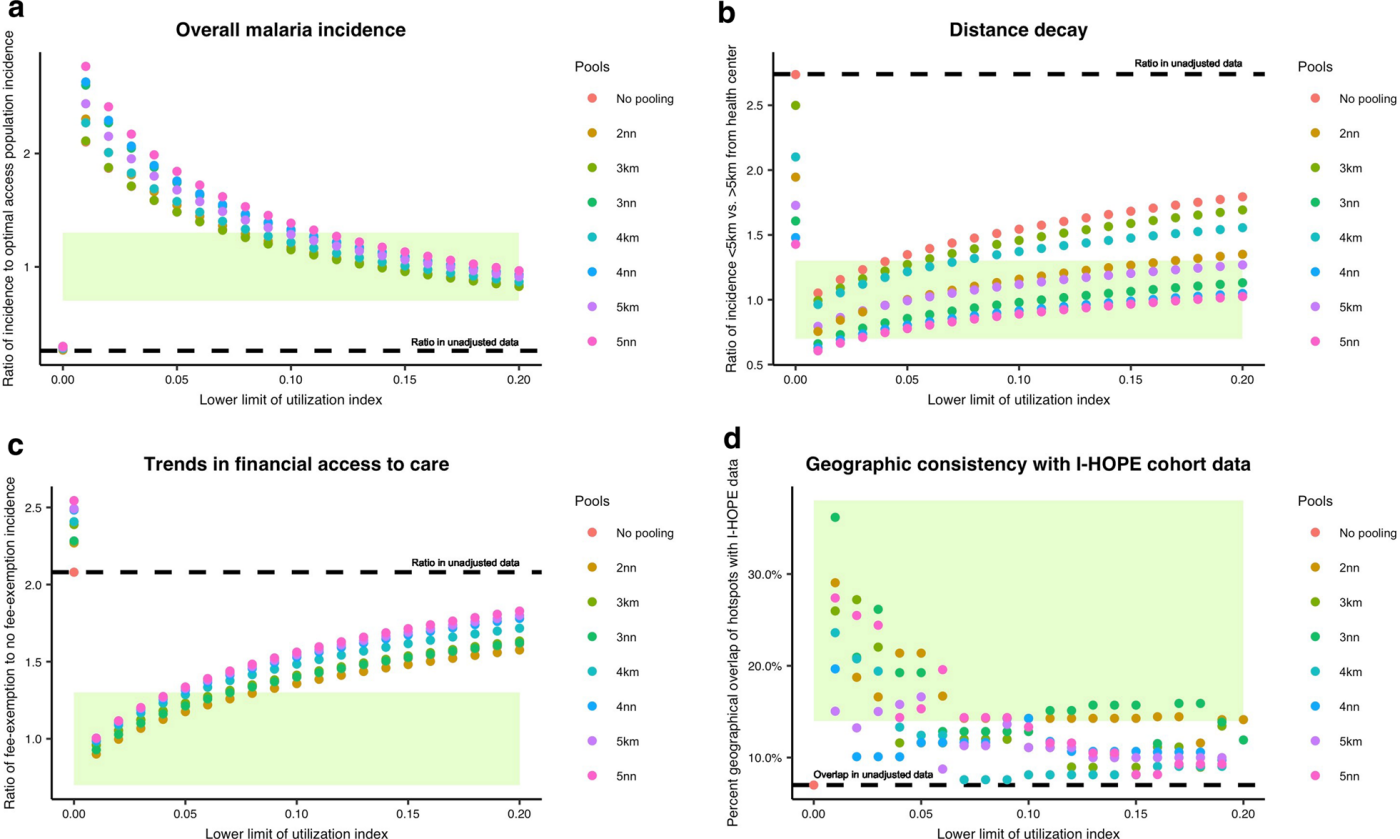
Summary results for the four evaluation criteria in unadjusted data and all adjusted malaria datasets. Each dot represents the metric of interest in one set of adjusted data, and colors represent the pooling strategy (e.g. 2 nn = pooling of Fokontany with its 2 nearest neighbors; 3 km = pooling with neighbors within 3 km). The dashed line shows values for the unadjusted dataset. Shaded green areas show target ranges of each evaluation criteria as described in Table 1. **a** Overall malaria incidence: ratio of malaria in adjusted dataset to malaria in optimal access areas. Values closer to 1 mean better performance. **b** Distance decay: ratio of incidence in Fokontany less than 5 km from a health center to incidence in Fokontany more than 5 km from a health center. Values closer to 1 mean better performance. **c** Trends in financial access to care: ratio of average monthly incidence in fee-exempt to non-fee-exempt populations in each adjusted dataset. Values closer to 1 mean better performance. **d** Geographic consistency with IHOPE cohort data: percent of overlap between hotspots of fever identified in the IHOPE cohort study data and malaria incidence in each adjusted dataset. Greater values mean better performance. Equivalent plots including analyses for children under 5 years can be found Additional file 1: Figure S2

**Fig. 5:**
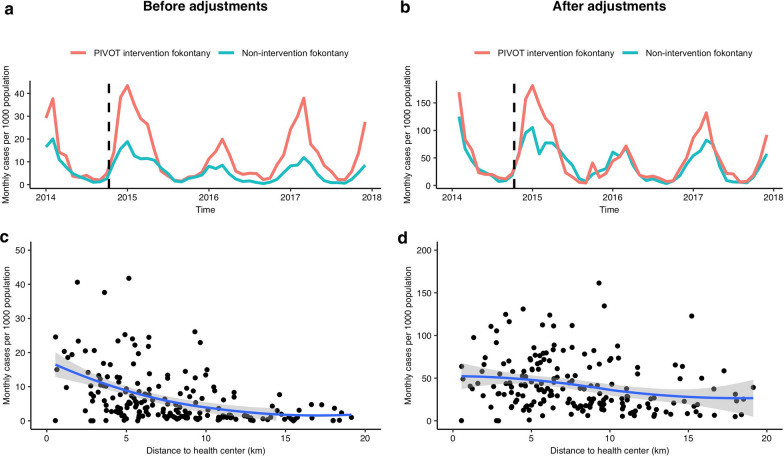
Temporal and geographic patterns in malaria, before and after adjustments. The top two panels show the average monthly cases per 1000 population over time, with colors representing the PIVOT intervention (orange) and non-intervention (teal) Fokontany, **a** before and **b** after adjustments in the most plausible dataset. The vertical dashed lines indicate the date (October 2014) when user fees were removed from health centers in PIVOT intervention Fokontany. The bottom two panels show the average monthly malaria cases per 1000 population in each Fokontany by distance to the nearest health center, **c** before and **d** after adjustments for health care access. Solid lines are the smoothed conditional means (LOESS method) and grey areas are the 95% confidence interval around the mean. Equivalent plots including only children under 5 years can be found in Additional file 1: Figure S5

Of the 168 adjusted datasets evaluated for individuals of all ages (Fig. 4), only one dataset fulfilled the four criteria described above (Table 3) and 86 datasets fulfilled three of the four criteria. Every pooling group and lower limit of utilization index was represented among the datasets that fulfilled three but not four criteria. We observed a clear trade-off in the adjusted datasets for the different evaluation criteria. Setting the lower limit for the utilization index at lower values (e.g. 0.01–0.07) resulted in better corrections for financial and geographic trends but overall incidence was above acceptable thresholds (Fig. 4, Additional file 1: Figure S2). In contrast, setting the lower limit for the utilization index at higher values (e.g. ≥ 0.09) resulted in overall incidence closest to incidence in the Fokontany with optimal access to care, but there remained important bias due to financial and geographic access (Fig. 4). The most plausible dataset was obtained using a lower limit of 0.08 for the health care utilization index in the benchmark multiplier method, and pooling Fokontany with two nearest neighbors. Figure 5 shows how the adjustment in this dataset improved geographic and temporal patterns in malaria incidence, reducing the apparent difference between Fokontany inside and outside of PIVOT intervention following user-fee removal, and removing the distance decay observed in the unadjusted dataset.

**Table 3:** Summary results for the four evaluation criteria in unadjusted data and best-performing adjusted malaria dataset for individuals of all ages

Dataset	Ratio of average incidence in dataset to incidence in optimal access areas	Ratio of incidence < 5 km to > 5 km from a health center	Ratio of incidence in fee-exemption to non-fee-exemption areas in dataset	% of hotspot clusters overlapped between dataset and IHOPE cohort data
Unadjusted register data	0.26	2.74	2.08	7
2 nearest neighbors, utilization index 0.08–1	1.29	1.10	1.29	14

An equivalent table for children under 5 years can be found in Additional file 1: Table S2

For children under five, 13 datasets satisfied the four criteria. The lower limits of utilization were higher than for individuals of all ages, ranging from 0.14 to 0.2 (Additional file 1: Table S2, Figure S2). Similar to the trends among all individuals, setting the lower limit of the utilization index at lower values (0–0.15) improved corrections for financial and geographic trends, but resulted in unacceptably high overall incidence. Datasets with high utilization index values (0.15–0.2) and low pooling groups (2–3 nearest neighbors) performed best overall. The most plausible dataset was obtained using a health care utilization index rescaled from 0.19 to 1 in the benchmark multiplier method, and pooling Fokontany with three nearest neighbors.

### The hidden burden of malaria and its local spatio-temporal dynamics in a rural health district

Using adjusted incidence estimates from the most plausible dataset, we estimated that the number of malaria cases diagnosed via passive surveillance in Ifanadiana from January 2014 to December 2017 represented only 21% of the total number of cases that could have occurred among all individuals during the study period, and 32% among children under 5 (Table 4). Average adjusted malaria incidence was estimated at 518 per 1000 population per year for individuals of all ages (43 per 1000 per month) and 733 per 1000 population per year for children under 5 (61 per 1000 per month). Average adjusted malaria incidence per month was nearly four times higher during the high transmission season (70 per 1000) than during the low transmission season (18 per 1000).

**Table 4:** Average monthly incidence of malaria for individuals of all ages in Ifanadiana from unadjusted and adjusted data, by transmission season and PIVOT intervention area, in cases per 1000 population

Year	Unadjusted data					Adjusted data				
	Malaria season (all Fokontany)		PIVOT intervention Fokontany (all seasons)		Overall	Malaria season (all Fokontany)		PIVOT intervention Fokontany (all seasons)		Overall
2014	High	17	Intervention	14	11	High	84	Intervention	60	52
	Low	4	Non-intervention	8		Low	23	Non-intervention	46	
2015	High	17	Intervention	14	10	High	84	Intervention	61	51
	Low	3	Non-intervention	7		Low	22	Non-intervention	44	
2016	High	9	Intervention	9	6	High	47	Intervention	31	30
	Low	3	Non-intervention	4		Low	12	Non-intervention	29	
2017	High	15	Intervention	16	9	High	71	Intervention	55	44
	Low	4	Non-intervention	5		Low	16	Non-intervention	37	

An equivalent table for children under 5 years can be found in Additional file 1: Table S3

**Fig. 6:**
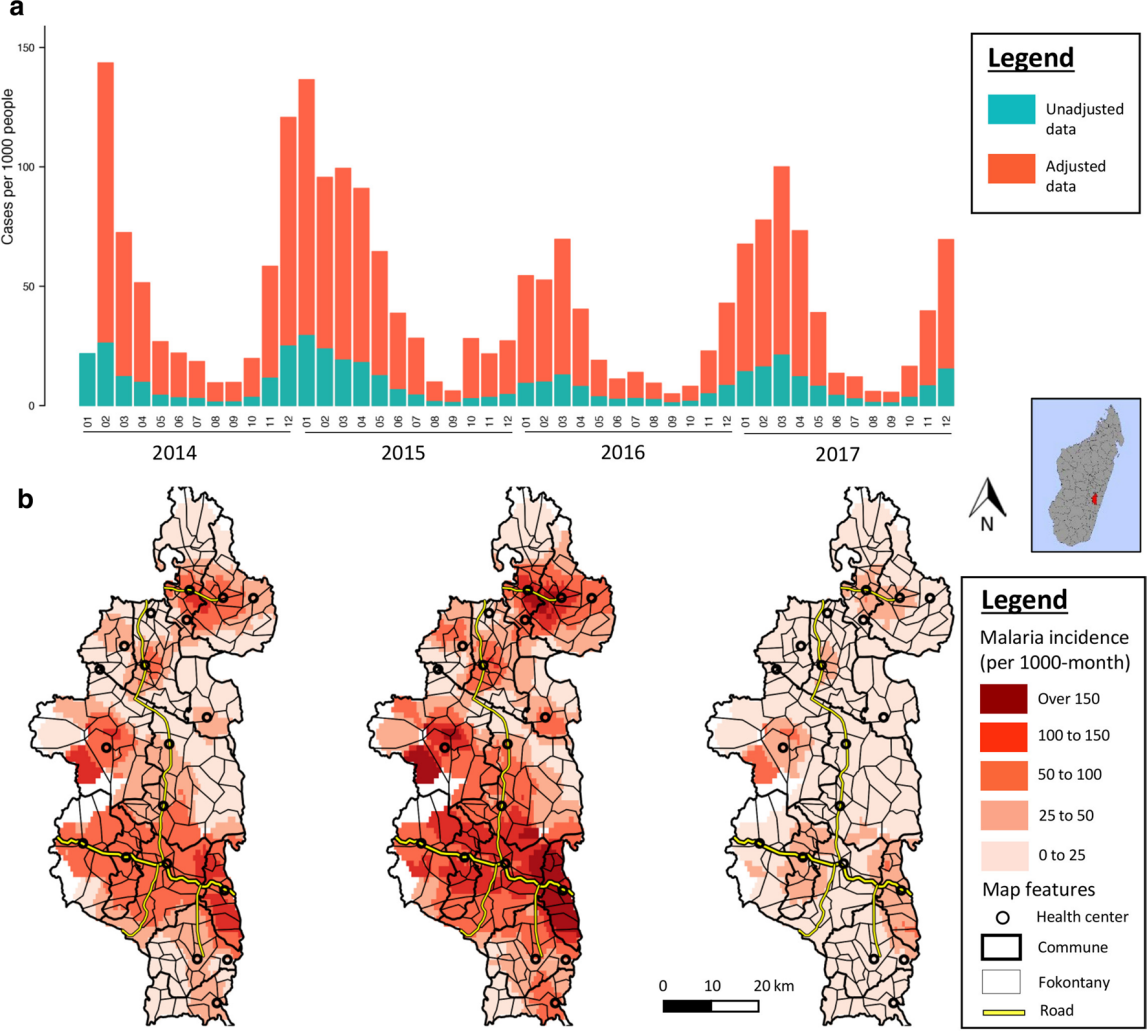
Temporal and spatial dynamics of adjusted monthly malaria incidence in Ifanadiana, 2014–2017. **a** Average number of new cases per 1000 population of all ages per month in the most plausible adjusted dataset (orange) and before adjustment (teal). An equivalent plot including only children under five can be found in Additional file 1: Figure S6. **b** Geographic distribution of malaria, averaged over all months (left), high season months (December to May; center), and low season months (June to November; right). Color gradient represents average monthly malaria incidence per 1000 population. Equivalent plots of spatial distribution in unadjusted health center register data are included in Additional file 1: Figure S4

Temporal dynamics in the adjusted dataset showed a decrease in malaria incidence from 2014–2015 (613 cases per 1000 per year) to 2016–2017 (441 cases per 1000 per year), with peaks in monthly incidence decreasing from almost 150 to about 100 cases per 1000 respectively (Fig. 6a). This trend is observed to a lesser degree in the unadjusted data, but when unadjusted data is disaggregated by intervention area, incidence in PIVOT intervention areas appear to have increased since 2014, likely due to increased access to care in these areas. After adjustments, the average monthly incidence of malaria is higher overall and more stable over time as well as between intervention and non-intervention areas due to adjustments for changing health care utilization (Fig. 6a).

By gathering patient-level data and removing systematic biases in the dataset, the spatial resolution of passive malaria surveillance in Ifanadiana district was improved by a factor of 10.2, from an average of 209.0 km^2^ (health center catchment) to 20.4 km^2^ (Fokontany area) per data point. Geographic distribution in the adjusted dataset revealed clusters of high incidence in low elevation areas in the northeast and southeast of the district (Fig. 6b). In addition, another high incidence cluster was observed in the western part of the district, at higher elevation and close to the limits of Ranomafana National Park. These high transmission clusters were stable across transmission seasons (Fig. 6b) and years (Additional file 1: Figure S3). In addition, 5% of Fokontany in Ifanadiana district had an average incidence higher than 100 cases per 1000 per month, distributed mostly in the central and southern part of the district (Fig. 6b). In comparison, the unadjusted dataset only revealed areas of high incidence in very close proximity to health centers along the main paved road and with user-fee exemptions in place (Additional file 1: Figure S4), missing most relevant transmission areas. Detailed spatio-temporal dynamics of malaria per month, from both the unadjusted and the most plausible adjusted dataset can be visualized in Additional file 3: Video S1.

## Discussion

Despite the increasing use of disease modeling and precision health mapping to inform national or regional health planning, their application remains scarce at the local level, where intervention efforts actually take place. This is especially true in rural areas of sub-Saharan Africa where the burden of infectious diseases is the highest. Improving the quality of routine surveillance data is critical for identifying at-risk populations and targeting resources in order to achieve universal access to diagnostics and treatment, which could contribute to the elimination of endemic diseases like malaria [51]. Here, we propose a method to improve existing passive surveillance data using models of health care utilization in order to produce more realistic estimates of local disease incidence over space and time. Using the example of malaria in a poor rural district of Madagascar, we show that adjusted incidence estimates were less biased by differences in financial and geographic access to health care between populations. We estimated that passive surveillance in Ifanadiana could have missed about 4 in every 5 cases of malaria and 2 out of every 3 cases among children under five. Removing systematic biases in reporting allowed us to downscale estimates of malaria incidence, improving their spatial resolution about ten-fold and revealing local heterogeneities in malaria transmission at scales that can be actionable by district health actors.

Passive surveillance systems are a cornerstone of many disease control programs because they are relatively inexpensive and can efficiently cover large geographic areas. When access to health care is relatively homogenous in a country, variations in incidence across districts help control programs identify those with higher transmission and allocate resources accordingly [52, 53]. However, at the local level of a health district these systems are systematically biased towards areas of good health care access (e.g. near health centers), preventing the implementation of geographically targeted interventions in areas of high transmission. Active surveillance systems, on the other hand, can capture a significantly higher proportion of cases and produce more accurate incidence estimates. Unfortunately, in the case of malaria they are too expensive to be used routinely in areas of high transmission, and the results cannot be extrapolated to detect variations in malaria in regions outside of the study area or period [13–18]. Thus, our study fills a significant gap for malaria surveillance, which could be applicable to other diseases. Using existing passive surveillance data, we were able to produce spatially-explicit estimates of malaria incidence for every community within a health district over time, identifying hotspots of transmission in communities with poor health care access that were previously invisible from passive surveillance. This could help inform local program implementation in high transmission settings without requiring extensive resources.

Without improvements to passive surveillance strategies, countless preventable cases and deaths of malaria may continue to take place and go unnoticed, which could undermine goals set for a 90% reduction in malaria mortality and the elimination in at least 35 countries by the year 2030 [12]. Using only routine health facility data, our results suggest that only 21% of malaria cases were detected by the passive surveillance system in our study area. This is consistent with findings from other settings where active and passive malaria surveillance methods were compared. For example, a study in rural Kenya found that the incidence of malaria in children was over three times higher when active surveillance was used compared to passive surveillance [15]. A similar study in central India reported that malaria incidence was almost eight times higher when calculated using active rather than passive surveillance data [16]. In 2012, the World Health Organization estimated that only 14% of malaria cases globally were captured by routine surveillance [17]. Our setting is representative of many rural areas in the developing world, where rough landscapes, poor road infrastructures and sparsely distributed populations make it difficult for patients to access health centers. More than 95% of paths were only accessible by foot, and three fourths of the population live more than an hour's walk of a public health center [34, 35], a commonly accepted threshold of low geographic access [54–57]. All these factors can lead to significant underreporting of malaria, at levels compatible with estimates presented here.

Although our study was retrospective and we had to collect information directly from paper registers, which was extremely time and resource consuming, this approach could be scaled-up in the future to other settings and diseases that rely on passive surveillance. Indeed, a push for electronic data collection to improve health information systems is underway at health care facilities of developing countries, with the current scale-up of the open source DHIS2 (District Health Information Software) [58] among other e-health platforms. These platforms are increasingly using mobile tools for registering cases and track patient-level data at different levels of care in order to move towards electronic surveillance of communicable diseases [59]. Yet, a recent review showed that studies seldom used routine data to characterize spatio-temporal risk of malaria at subnational scales due to limited quality and systematic biases, and none used routine health facility data at a finer scale than the facility’s catchment [60]. Integration of the methods presented here into electronic surveillance systems would allow the use of these granular data, requiring little additional information and straightforward geostatistical techniques. Data on stocks of malaria supplies is commonly available as part of the national HMIS. Moreover, a local characterization of the main drivers of health care utilization over space and time can be obtained elsewhere using available maps of geographic accessibility to health facilities (available at the global level at a 1 km × 1 km resolution [61]), data from patients coming to health facilities for diseases other than malaria, and institutional knowledge about the timing and geographic extent of interventions that can have major impacts on health care utilization (e.g. user fee exemptions, health insurance, etc.).

The level of granularity and timeliness of data that the scale up of e-health platforms offer when compared with traditional health management and information systems (e.g. paper-based registries, monthly aggregation in electronic databases) opens new possibilities for disease control, which are still largely unexplored. Fine-scale estimates of malaria spatio-temporal variations using methods presented here can then be used to characterize local socio-economic and environmental drivers of malaria risk, paving the way to the development of early warning or forecasting systems that could further guide local malaria control. Malaria heterogeneity and its drivers are commonly modelled at the national and regional level [62–64], but malaria can have extensive spatial variability in relatively small areas [42, 43, 65]. After adjustment, we observed significant spatial variations in malaria incidence in communities across the district, with 7% the population living in areas where annual incidence was twice the district’s average, as well as multiple short-term, localized hotspots during the study period (Additional file 3: Video S1). Fine-scale variations in socio-demographic and behavioral factors can influence malaria risk in remote communities [66] or affect adherence to malaria control programs [67]. Moreover, local variations in environmental factors such as temperature, rainfall, land cover, and altitude have been shown to influence malaria geographic distribution [68–70]. Therefore, integration of feedback loops between disease modelling approaches and e-health surveillance platforms at these local scales could help to (1) target efforts and plan resources necessary ahead of time for specific areas and periods, reducing stock-outs and increasing case detection; and (2) implement additional control activities that are predicted to minimize transmission within a health district.

This study had several limitations. First, there was no active surveillance campaign during the study period that could serve as a true comparison point for selecting the most plausible set of estimates. As an alternative, we compared adjusted estimates with areas within the district that had optimal access to care and therefore were assumed to have missed few malaria cases. However, if these areas were not representative of overall malaria incidence due to heterogeneities, this could have resulted in an under- or overestimation. Second, many of the most remote Fokontany did not report any malaria cases even during high transmission seasons. To allow for adjustments and minimize underestimation of malaria in these remote populations, we pooled these Fokontany with their nearest neighbors, but this likely reduced the spatial precision of our estimates. Third, even though we correct for health care access, there were still some patterns in the adjusted datasets (e.g. higher incidence around PIVOT-supported health centers), which could suggest an influence of unmeasured factors not accounted for in our analyses. Finally, although data on RDT stock-outs was available, underreporting of the number of days without stocks in some health centers could have led to artificially low malaria estimates. Despite its limitations, we are not aware of any other study that has attempted to systematically address sources of malaria underreporting to generate realistic incidence estimates from passive surveillance systems at such local scales.

## Conclusion

Although passive surveillance at health facilities remains the prevailing surveillance system for many endemic diseases in the developing world, systematic biases in these data prevent their use to inform local disease control programs within health districts. By adjusting for health care access and other known sources of underreporting, we show that passive surveillance can be used to obtain realistic estimates of malaria dynamics with a level of spatial resolution that is locally actionable. Future research should assess whether such methods can be scaled-up and integrated with e-health platforms currently being deployed.

## Supplementary Information

**Additional file 1.** It contains 6 additional figures and 2 additional tables, with results for children under 5 years and other additional information.

**Additional file 2.** STROBE checklist. The STROBE statement is a checklist of 22 items considered essential for good reporting of observational studies. STROBE, Strengthening the Reporting of Observational Studies in Epidemiology.

**Additional file 3: Video S2.** Video of malaria spatio-temporal dynamics. It shows geographic changes in monthly malaria incidence in Ifanadiana district (unadjusted and adjusted estimates from the most plausible dataset). For reference, removal of user fees in the initial HSS intervention catchment (in red) took place in October 2014, and was expanded to one additional commune in October 2017.

## Data Availability

Data may be made available upon request at research@pivotworks.org.
