# Supplementary material for: Estimating the local spatio‐temporal distribution of malaria from routine health information systems in areas of low health care access and reporting
Source: Int J Health Geogr. 2021 Feb 12;20:8. doi: 10.1186/s12942-021-00262-4 (PMC7879399; doi:10.1186/s12942-021-00262-4)
Supplement: Supplementary file 1 — Additional file 1. It contains 6 additional figures and 2 additional tables, with results for children under 5 years and other additional information. [file 12942_2021_262_MOESM1_ESM.docx]

**Estimating the local spatio-temporal distribution of malaria from routine health information systems in areas of low health care access and reporting**

**- Supplementary information -**

Elizabeth Hyde^1^, Matthew H. Bonds^2,3^, Felana A. Ihantamalala^2,3^, Ann C. Miller^2^, Laura F. Cordier^3^, Benedicte Razafinjato^3^, Herinjaka Andriambolamanana^3^, Marius Randriamanambintsoa^4^, Michele Barry^5,6^, Jean-Claude Andrianirinarison^7,8^, Mauricette A. Nambinisoa^7^, Andres Garchitorena^3,9*^

^1^ Stanford University School of Medicine, Stanford, CA, USA

^2^ Department of Global Health and Social Medicine, Harvard Medical School, Boston, USA

^3^ NGO PIVOT, Ranomafana, Madagascar

^4^ Direction de la Démographie et des Statistiques Sociales, Institut National de la Statistique, Antananarivo, Madagascar

^5^ Center for Innovation in Global Health, Stanford University, Stanford, CA, USA

^6^ Office of the Dean, Stanford University School of Medicine, Stanford, CA, USA

^7^ Ministry of Public Health, Antananarivo, Madagascar

^8^ National Institute of Public Health, Antananarivo, Madagascar

^9^ MIVEGEC, Univ. Montpellier, CNRS, IRD, Montpellier, France

*Corresponding author: andres.garchitorena@gmail.com

**Table S1.** Multivariate model results of per-capita outpatient visits at Ifanadiana public health centers (PHC) in 2014-2017, excluding malaria cases (generalized linear mixed model with random intercept at the health center closest to the Fokontany of residence).

| **Variable** | **Odds Ratio (95% CI)** |
| --- | --- |
| Intercept (Visits per capita-month) | 0.015 (0.008-0.03) |
| **Geographic factors** |  |
| Network distance to PHC (10km, linear)^a^ | 0.095 (0.091-0.1) |
| Network distance to PHC (10km, quadratic)^a^ | 1.261 (1.226-1.297) |
| **Health system factors** |  |
| Number of health staff | 1.043 (1.037-1.049) |
| Major PHC (vs. basic PHC) | 3.16 (1.421-7.024) |
| PIVOT initial catchment (vs. outside) | 0.641 (0.611-0.672) |
| **Impact of HSS programs** |  |
| User fee exemption program | 1.18 (1.145-1.215) |
| Interaction with Distance to PHC (10km)^a^ | 1.44 (1.415-1.466) |
| Community health program | 1.112 (1.087-1.137) |
| Interaction with Distance to PHC (10km)^a^ | 1.127 (1.109-1.145) |
| **Underlying trends** |  |
| Linear trend (year) | 0.988 (0.981-0.995) |
| Seasonal trend^b^ | 1.031 (1.024-1.038) |
| Lagged trend (1 month lag)^c^ | 1.65 (1.637-1.663) |

^a^ The variable network distance represents tens of kilometers (distance in km*10^-1^) to facilitate interpretation of coefficients and enable model convergence

^b^ Seasonal trend was constructed as [sin(2π(Month_i_ + θ/12],where θ was the horizontal shift that best fit the data.

^c^ Lagged trend transformed into visits per capita-year to allow interpretation of results


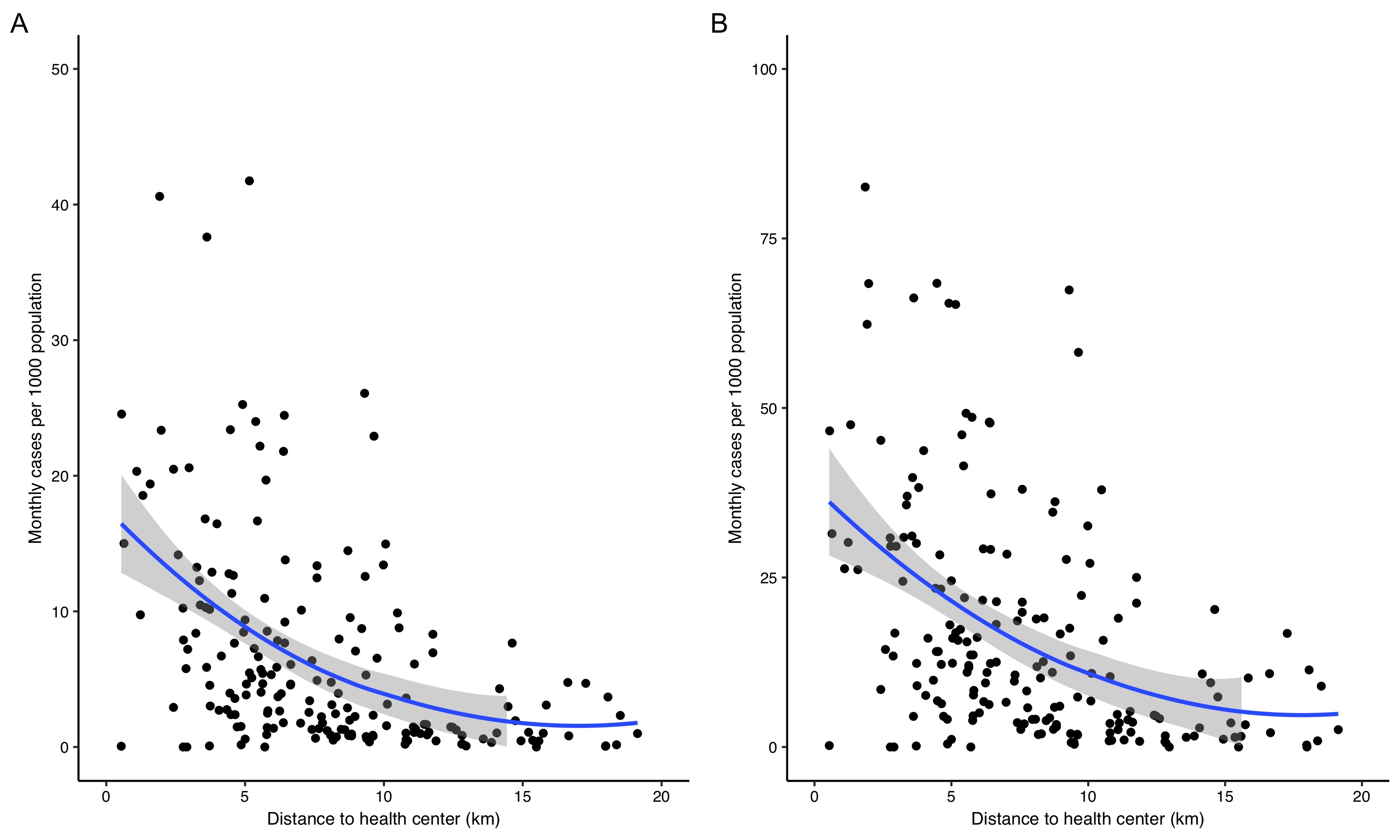


**Figure S1. Distance decay in unadjusted average monthly malaria incidence in Ifanadiana, 2014-2017.** (A) For all individuals and (B) for children under 5 years. Dots represent average monthly malaria incidence for each of the 195 Fokontany in Ifanadiana. Solid lines are the smoothed conditional means (LOESS method) and grey areas are the 95% confidence interval around each the mean. To improve visualization, 1 dot in panel B was removed.


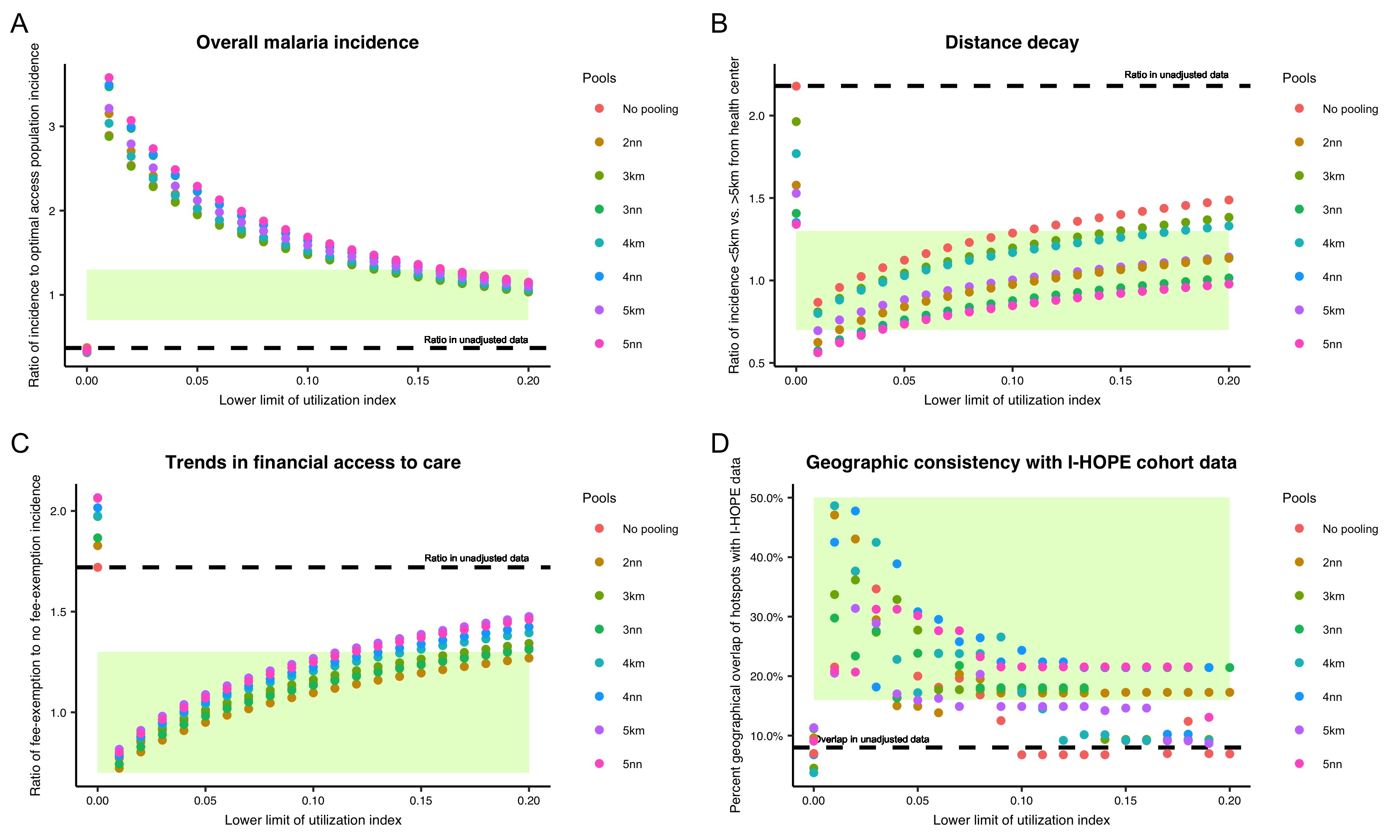


**Figure S2. Summary results for the four evaluation criteria in unadjusted data and all adjusted malaria datasets in children under five.** Each dot represents the metric of interest in one set of adjusted data, and colors represent the pooling strategy. The dashed line shows values for the unadjusted dataset. Shaded green areas show target ranges of evaluation criteria. (A) Overall malaria incidence: ratio of malaria in adjusted dataset to malaria in optimal access areas. Values closer to 1 mean better performance. (B) Distance decay: ratio of incidence in Fokontany less than 5 km from a health center to incidence in Fokontany more than 5 km from a health center. Values closer to 1 mean better performance. (C) Trends in financial access to care: ratio of average monthly incidence in fee-exempt to non-fee-exempt populations in each adjusted dataset. Values closer to 1 mean better performance. (D) Geographic consistency with I-HOPE cohort data: percent of overlap between hotspots of fever identified in the I-HOPE cohort study data and malaria incidence in each adjusted dataset. Greater values mean better performance.


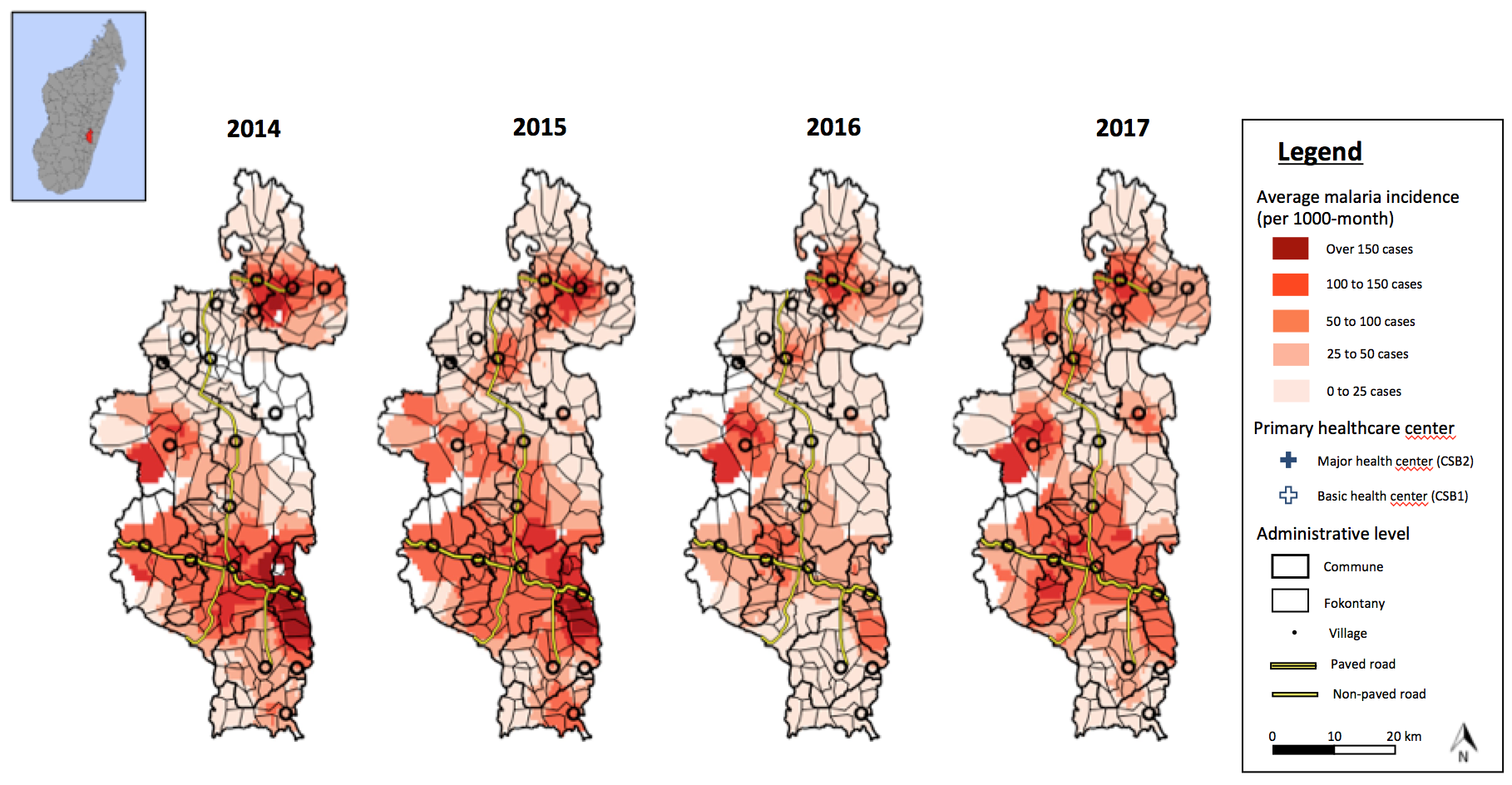


**Figure S3. Average adjusted monthly malaria incidence in Ifanadiana for each year of the study period, 2014-2017.** Color gradient represents average monthly malaria incidence per 1000 population.


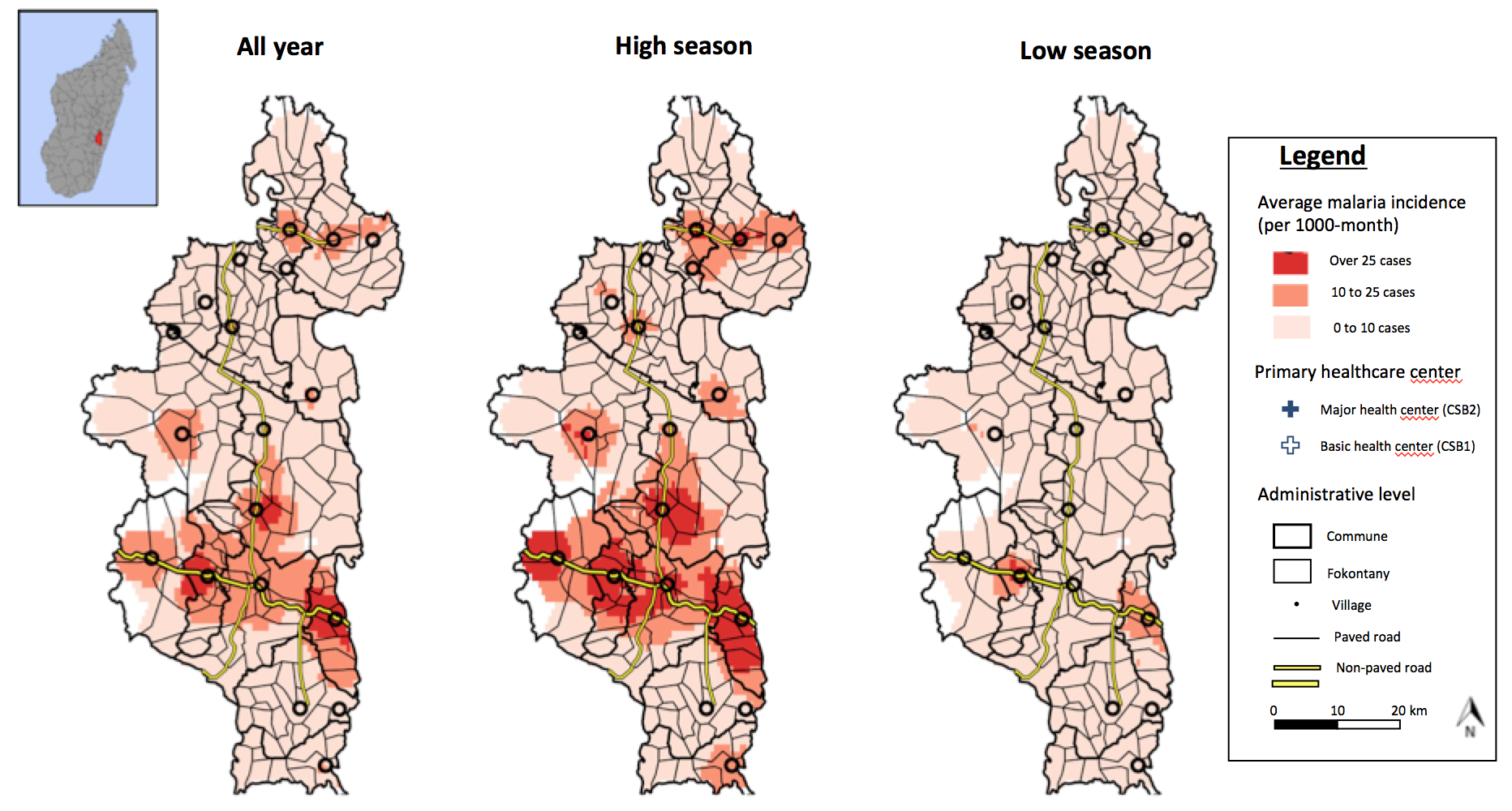


**Figure S4. Geographic dynamics of unadjusted monthly malaria incidence by malaria season in Ifanadiana, 2014-2017.** Geographic distribution of malaria, averaged over all months (left), high season months (December to May; center), and low season months (June to November; right). Color gradient represents average monthly malaria incidence per 1000 population.

**Table S2. Summary results for the four evaluation criteria in unadjusted data and best-performing adjusted malaria datasets for children under five^1^.**

| **Dataset** | **Ratio of average incidence in dataset to incidence in optimal access areas** | **Ratio of incidence**  **< 5km to > 5km from a health center** | **Ratio of incidence in fee-exemption to non-fee-exemption areas in dataset** |  | **% of hotspot clusters overlapped between dataset and I-HOPE cohort data** |
| --- | --- | --- | --- | --- | --- |
| Unadjusted register data | 0.37 | 2.18 | 1.72 |  | 8% |
| 2 nearest neighbors,  utilization index 0.14 – 1 | 1.27 | 1.05 | 1.18 |  | 17% |
| 2 nearest neighbors,  utilization index 0.15 – 1 | 1.22 | 1.07 | 1.20 |  | 17% |
| 2 nearest neighbors,  utilization index 0.16 – 1 | 1.18 | 1.08 | 1.21 |  | 17% |
| 2 nearest neighbors,  utilization index 0.17 – 1 | 1.14 | 1.09 | 1.23 |  | 17% |
| 2 nearest neighbors,  utilization index 0.18 – 1 | 1.10 | 1.11 | 1.24 |  | 17% |
| 2 nearest neighbors,  utilization index 0.19 – 1 | 1.07 | 1.12 | 1.26 |  | 17% |
| 2 nearest neighbors,  utilization index 0.2 – 1 | 1.03 | 1.13 | 1.27 |  | 17% |
| Neighbors within 3km, utilization index 0.14 – 1 | 1.26 | 1.28 | 1.25 |  | 9% |
| 3 nearest neighbors,  utilization index 0.16 – 1 | 1.28 | 0.97 | 1.25 |  | 21% |
| 3 nearest neighbors,  utilization index 0.17 – 1 | 1.24 | 0.98 | 1.27 |  | 21% |
| 3 nearest neighbors,  utilization index 0.18 – 1 | 1.20 | 0.99 | 1.28 |  | 21% |
| **3 nearest neighbors,**  **utilization index 0.19 – 1** | **1.16** | **1.00** | **1.30** |  | **21%** |
| Neighbors within 4km,  utilization index 0.14 – 1 | 1.29 | 1.25 | 1.29 |  | 10% |

^1^Color code for dataset performance: orange=poor; yellow=acceptable; light green=good; green=exceptional

**Table S3. Average monthly incidence of malaria for children under five in Ifanadiana from unadjusted and adjusted data, by transmission season and PIVOT intervention area, in cases per 1000 population.**

| **Year** | **Unadjusted data** | | | | | **Adjusted data** | | | | |
| --- | --- | --- | --- | --- | --- | --- | --- | --- | --- | --- |
|  | **Malaria season (all Fokontany)** | | **PIVOT intervention Fokontany (all seasons)** | | **Overall** | **Malaria season (all Fokontany)** | | **PIVOT intervention Fokontany (all seasons)** | | **Overall** |
| 2014 | High | 36 | Intervention | 31 | 23 | High | 114 | Intervention | 82 | 72 |
|  | Low | 11 | Non-intervention | 19 |  | Low | 35 | Non-intervention | 65 |  |
| 2015 | High | 41 | Intervention | 31 | 24 | High | 127 | Intervention | 89 | 76 |
|  | Low | 8 | Non-intervention | 19 |  | Low | 32 | Non-intervention | 67 |  |
| 2016 | High | 19 | Intervention | 18 | 13 | High | 65 | Intervention | 45 | 43 |
|  | Low | 7 | Non-intervention | 10 |  | Low | 20 | Non-intervention | 41 |  |
| 2017 | High | 28 | Intervention | 28 | 18 | High | 91 | Intervention | 72 | 58 |
|  | Low | 8 | Non-intervention | 13 |  | Low | 25 | Non-intervention | 50 |  |


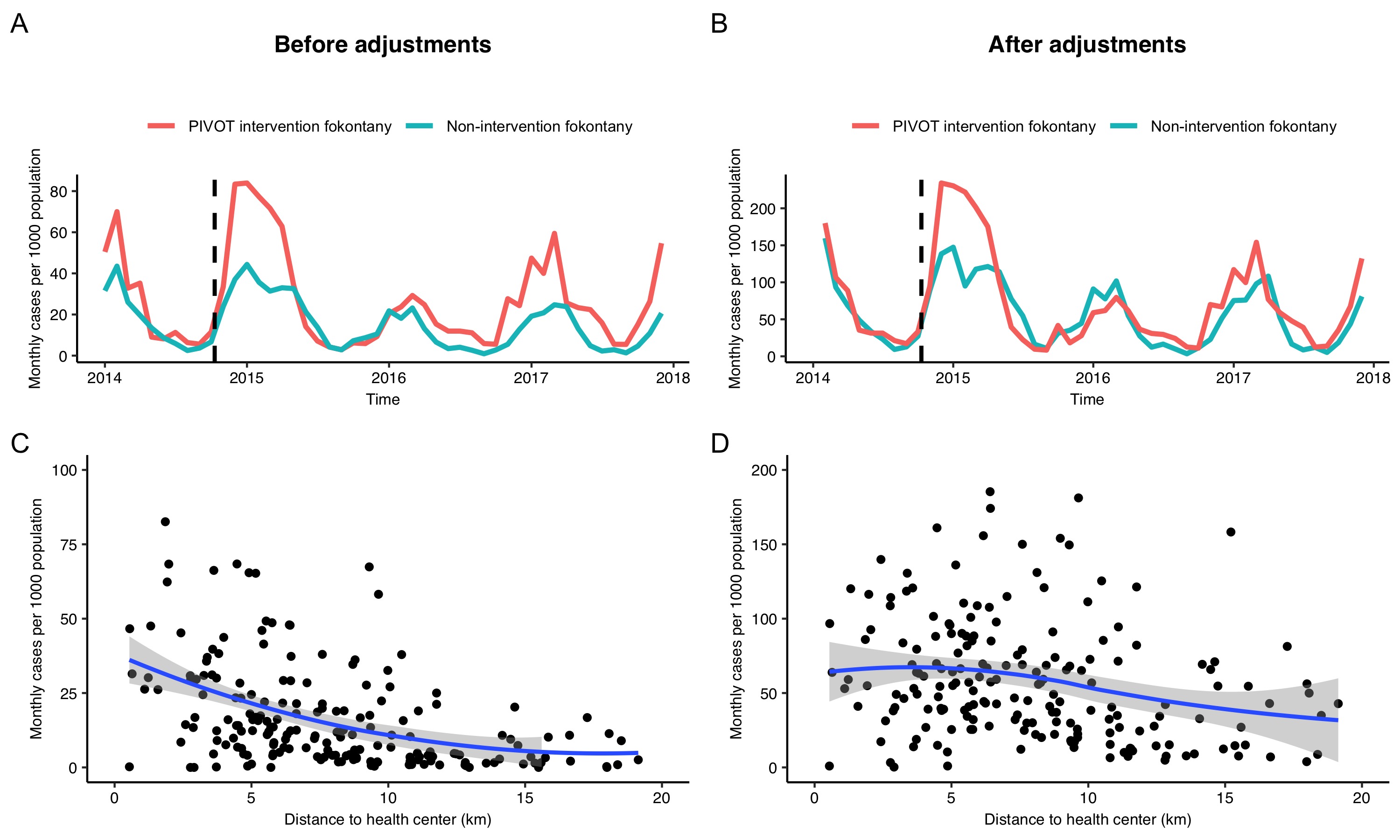


**Figure S5. Temporal and geographic patterns in malaria, before and after adjustments, among children under five.** The top two panels show the average monthly cases per 1000 population over time, with colors representing the PIVOT intervention (orange) and non-intervention (teal) Fokontany, (A) before and (B) after adjustments in the most plausible dataset. The vertical dashed lines indicate the date (October 2014) when user fees were removed from health centers in PIVOT intervention Fokontany. The bottom two panels show the average monthly malaria cases per 1000 population in each Fokontany by distance to the nearest health center, (C) before and (D) after adjustments for health care access. Solid lines are the smoothed conditional means (LOESS method) and grey areas are the 95% confidence interval around the mean. To improve visualization, 1 dot was removed from panel C.


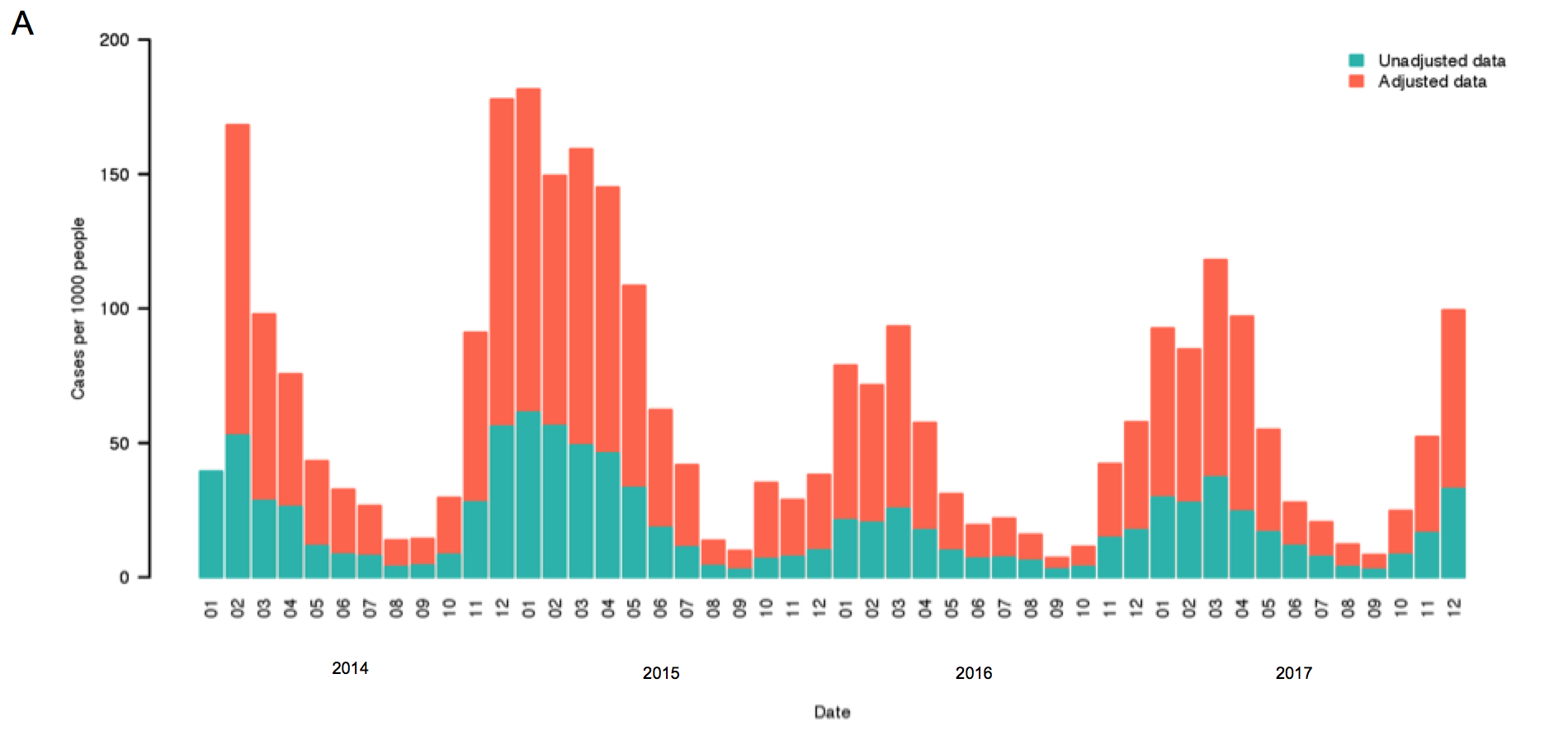


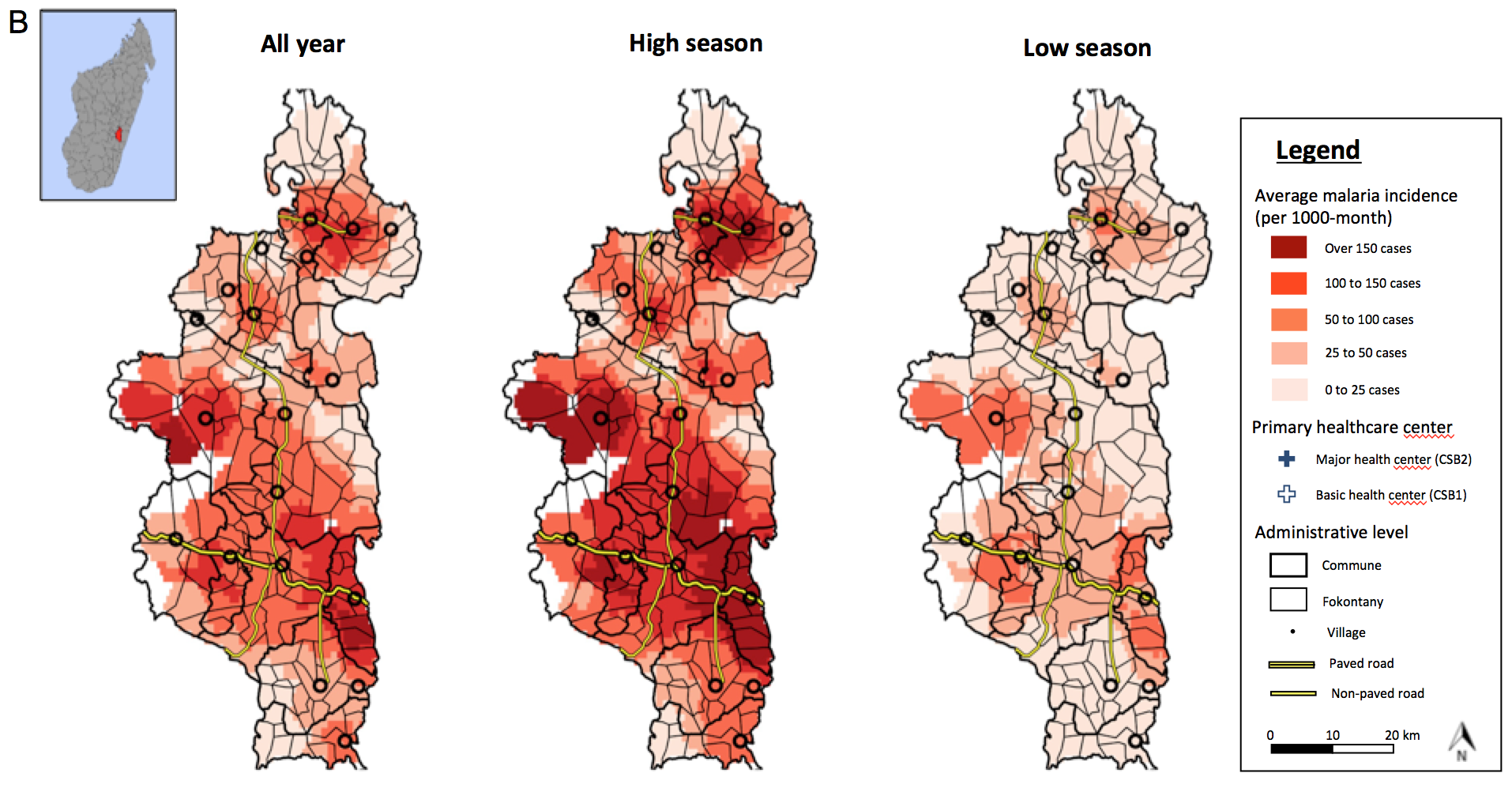


**Figure S6: Temporal and spatial dynamics of adjusted monthly malaria incidence among children under five in Ifanadiana, 2014-2017.** (A) Average number of new cases per 1000 population of all ages per month in the most plausible adjusted dataset (orange) and before adjustment (teal). (B) Geographic distribution of malaria, averaged over all months (left), high season months (December to May; center), and low season months (June to November; right). Color gradient represents average monthly malaria incidence per 1000 population.
