# Supplementary material for: Estimating the local spatio‐temporal distribution of malaria from routine health information systems in areas of low health care access and reporting
Source: Int J Health Geogr. 2021 Feb 12;20:8. doi: 10.1186/s12942-021-00262-4 (PMC7879399; doi:10.1186/s12942-021-00262-4)
Supplement: Supplementary file 2 — Additional file 2. STROBE checklist. The STROBE statement is a checklist of 22 items considered essential for good reporting of observational studies. STROBE, Strengthening the Reporting of Observational Studies in Epidemiology. [file 12942_2021_262_MOESM2_ESM.docx]

STROBE Statement—Checklist of items that should be included in reports of ***cohort studies***

|  | Item No | Recommendation | Page No |
| --- | --- | --- | --- |
| **Title and abstract** | 1 | (*a*) Indicate the study’s design with a commonly used term in the title or the abstract | a) Title: *Estimating the local spatio-temporal distribution of malaria from routine health information systems in areas of low health care access and reporting* |
|  |  | (*b*) Provide in the abstract an informative and balanced summary of what was done and what was found | b) The abstract provides this information |
| Introduction | | | |
| Background/rationale | 2 | Explain the scientific background and rationale for the investigation being reported | In paragraphs 1-4 of the Introduction section |
| Objectives | 3 | State specific objectives, including any prespecified hypotheses | In paragraph 4 of the Introduction section |
| Methods | | | |
| Study design | 4 | Present key elements of study design early in the paper | In paragraph 4 of the Introduction section and paragraph 8 of the Methodology section (Data analysis subsection) |
| Setting | 5 | Describe the setting, locations, and relevant dates, including periods of recruitment, exposure, follow-up, and data collection | In paragraphs 1-3 of the Methodology section (Study site subsection) and Figure 1 |
| Participants | 6 | (*a*) Give the eligibility criteria, and the sources and methods of selection of participants. Describe methods of follow-up | a) In paragraph 4-5 of the Methodology section (Data collection subsection) |
|  |  | (*b*) For matched studies, give matching criteria and number of exposed and unexposed | b) Not applicable |
| Variables | 7 | Clearly define all outcomes, exposures, predictors, potential confounders, and effect modifiers. Give diagnostic criteria, if applicable | In paragraphs 8-11 of the Methodology section (Data analysis subsection) |
| Data sources/ measurement | 8* | For each variable of interest, give sources of data and details of methods of assessment (measurement). Describe comparability of assessment methods if there is more than one group | In paragraph 4-7 of the Methodology section (Data collection subsections) |
| Bias | 9 | Describe any efforts to address potential sources of bias | In paragraph 15-20 of the Methodology section (Evaluation of model estimates subsection) |
| Study size | 10 | Explain how the study size was arrived at | In paragraph 4-5 of the Methodology section (Data collection subsection) |
| Quantitative variables | 11 | Explain how quantitative variables were handled in the analyses. If applicable, describe which groupings were chosen and why | In paragraphs 9-11 of the Methodology section (Data analysis subsection) |
| Statistical methods | 12 | (*a*) Describe all statistical methods, including those used to control for confounding | In paragraphs 8-14 of the Methodology section (Data analysis subsection) |
|  |  | (*b*) Describe any methods used to examine subgroups and interactions |  |
|  |  | (*c*) Explain how missing data were addressed |  |
|  |  | (*d*) If applicable, explain how loss to follow-up was addressed |  |
|  |  | (*e*) Describe any sensitivity analyses |  |
| Results | | |  |
| Participants | 13* | (a) Report numbers of individuals at each stage of study—eg numbers potentially eligible, examined for eligibility, confirmed eligible, included in the study, completing follow-up, and analysed | a) In paragraph 1 of the Results section  b-c) Flow diagram was not used (available in Garchitorena et al. Geographic Barriers to Achieving Universal Health Coverage in a rural district of Madagascar. MedRxiv. 2020.) |
|  |  | (b) Give reasons for non-participation at each stage |  |
|  |  | (c) Consider use of a flow diagram |  |
| Descriptive data | 14* | (a) Give characteristics of study participants (eg demographic, clinical, social) and information on exposures and potential confounders | In paragraph 1 of the Results section and Table 2 |
|  |  | (b) Indicate number of participants with missing data for each variable of interest |  |
|  |  | (c) Summarise follow-up time (eg, average and total amount) |  |
| Outcome data | 15* | Report numbers of outcome events or summary measures over time | In paragraphs 4-5 and Table 4 and Figure 5 of the Results section |

| Main results | 16 | (*a*) Give unadjusted estimates and, if applicable, confounder-adjusted estimates and their precision (eg, 95% confidence interval). Make clear which confounders were adjusted for and why they were included | a) In paragraphs 1-6 of the Results section, Tables 2-4, and Figures 4-6 |
| --- | --- | --- | --- |
|  |  | (*b*) Report category boundaries when continuous variables were categorized |  |
|  |  | (*c*) If relevant, consider translating estimates of relative risk into absolute risk for a meaningful time period |  |
| Other analyses | 17 | Report other analyses done—eg analyses of subgroups and interactions, and sensitivity analyses | Analysis for children under 5 years available in Additional file 1 |
| Discussion | | | |
| Key results | 18 | Summarise key results with reference to study objectives | In paragraph 1 of the Discussion section |
| Limitations | 19 | Discuss limitations of the study, taking into account sources of potential bias or imprecision. Discuss both direction and magnitude of any potential bias | In paragraph 6 of the Discussion section |
| Interpretation | 20 | Give a cautious overall interpretation of results considering objectives, limitations, multiplicity of analyses, results from similar studies, and other relevant evidence | In paragraph 2-3 of the Discussion section |
| Generalisability | 21 | Discuss the generalisability (external validity) of the study results | In paragraph 4-5 of the Discussion section |
| Other information | | | |
| Funding | 22 | Give the source of funding and the role of the funders for the present study and, if applicable, for the original study on which the present article is based | In Declarations section (Funding subsection) |
